# Optimum Carbon Fiber Reinforced Polymer (CFRP) Design for Flexural Strengthening of Cantilever Concrete Walls Using Artificial Neural Networks

**DOI:** 10.3390/polym17243300

**Published:** 2025-12-12

**Authors:** Gebrail Bekdaş, Ammar Khalbous, Sinan Melih Nigdeli, Ümit Işıkdağ

**Affiliations:** 1 Department of Civil Engineering, Istanbul University-Cerrahpasa, Istanbul 34320, Türkiye; bekdas@iuc.edu.tr (G.B.); ammar.khalbous@ogr.iuc.edu.tr (A.K.); 2 Department of Architecture, Mimar Sinan Fine Arts University, Istanbul 34427, Türkiye; umit.isikdag@msgsu.edu.tr

**Keywords:** carbon fiber reinforced polymer (CFRP), flexural strengthening, cantilever concrete walls, JAYA algorithm, artificial neural networks (ANN)

## Abstract

This study introduces a hybrid framework combining an Artificial Neural Network (ANN) with the Jaya optimization algorithm to predict the minimum Carbon Fiber Reinforced Polymer (CFRP) area required for flexural strengthening of reinforced concrete (RC) cantilever walls. A multilayer perceptron (MLP) network was trained on 500 Jaya-optimized design scenarios incorporating twelve design variables, including geometry, loads, and material properties. The ANN achieved high predictive accuracy, with R-values near 1.0 across training, validation, and testing phases. Five independent test cases yielded an average error of 3.69%, and 10-fold cross-validation confirmed model robustness (R = 0.9996). A global perturbation-based sensitivity analysis was also conducted to quantify the influence of each input parameter, highlighting wall length, moment demand, and concrete strength as the most significant features. This integrated ANN–Jaya model enables rapid, code-compliant CFRP design in accordance with ACI 318 and ACI 440.2R-17, minimizing material usage and ensuring economic and sustainable retrofitting. The proposed approach offers a practical, data-driven alternative to traditional iterative methods, suitable for application in modern performance-based structural engineering.

## 1. Introduction

**Figure 1:**
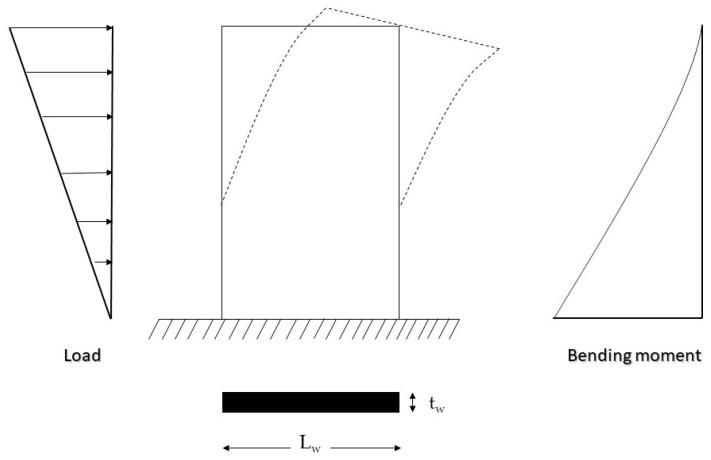
Lateral load (indicated by arrows), flexural deformation (indicated by dashed lines), and bending moment in a cantilever shear wall.

Cantilever concrete walls are essential structural elements in modern construction, providing critical resistance to lateral loads such as those induced by wind, seismic activity, and other forces. These walls, fixed at the base and free at the top, develop significant flexural moments and must possess adequate bending capacity. However, existing cantilever walls may require strengthening due to factors such as increased load demands, material degradation, or design deficiencies. The need for effective strengthening techniques is paramount to ensure the continued serviceability and safety of these structures [1]. The structural behavior of cantilever concrete shear walls under lateral loads is illustrated in Figure 1, including the triangular load distribution, lateral displacement due to flexural deformation, and the resulting bending moment diagram.

Traditional methods for strengthening reinforced concrete structures include adding external reinforcement, increasing the section size, or using steel plates. While these methods can be effective, they often require invasive interventions, are labor-intensive, and may not be practical in scenarios where minimizing downtime is critical. Furthermore, these methods can add substantial weight to the structure, which may be undesirable, particularly in seismic regions where additional weight can amplify seismic forces [1,2].

To address the structural deficiencies that arise due to aging, increased load demands, or design limitations in reinforced concrete (RC) elements, Carbon Fiber Reinforced Polymer (CFRP) has gained considerable attention as a high-performance strengthening material. Its unique combination of high tensile strength, lightweight characteristics, corrosion resistance, and ease of installation makes it an ideal solution for retrofitting concrete structures in a non-destructive manner. Unlike traditional strengthening methods, CFRP can be externally bonded to existing elements without major demolition or disruption to service, enabling efficient rehabilitation strategies [3].

Carbon-fiber reinforced polymers (CFRPs) exhibit much higher tensile strength and stiffness than glass-fiber (GFRP) or basalt-fiber (BFRP) composites [4]. In practice, CFRP’s modulus of elasticity and strength-to-weight ratio far exceed those of GFRP, while BFRP generally lies between them: for example, basalt-FRP sheets have reported tensile strengths of ~815–930 MPa and elastic moduli ~40–43 GPa [4,5], compared to carbon-fiber composites whose fibers typically reach ~200+ GPa modulus. All FRPs share inherent corrosion resistance (unlike steel) [6], but CFRP has superior chemical and UV stability relative to GFRP, and BFRP offers even better thermal and chemical durability and creep resistance [5,7]. In strength-retrofit applications, numerous studies confirm that adding CFRP laminates or wraps markedly increases the flexural capacity and ductility of RC elements. For instance, externally bonded CFRP to beams or walls significantly elevates load-carrying capacity, ultimate deflection, and energy dissipation; retrofitted RC beams are shown to carry higher loads and deform more before failure than identical unstrengthened beams [4]. Finite-element analyses similarly predict large gains, e.g., prestressed CFRP strips on hollow box girders substantially raised both yield and ultimate moment capacity [4]. In summary, compared to GFRP or BFRP, CFRP’s higher stiffness and strength and excellent durability make it especially effective for flexural strengthening of RC beams, walls, and slabs. This explains why CFRP retrofits consistently improve flexural strength, ductility, and deformation capacity in reinforced-concrete members, as needed for optimized design (e.g., via ANN prediction of required CFRP area) in cantilever shear walls and similar structures [4].

Experimental studies have confirmed that CFRP retrofitting substantially improves the flexural behavior of various RC members. Applications in slabs, cantilever beams, and wall-type elements have shown noticeable gains in load capacity and ductility, often transforming brittle failure modes into more ductile responses [3,8]. This is particularly relevant in the case of cantilever concrete walls, which are often exposed to high bending moments due to lateral loading conditions. When applied to the tension zone of such walls, externally bonded CFRP systems can effectively enhance both stiffness and moment capacity, enabling the structure to meet performance requirements without altering its original configuration [1,2]. A specific advantage of CFRP in wall strengthening is its adaptability to various configurations, such as continuous horizontal strips or discrete vertical bands, depending on structural demands. As demonstrated by Antoniades et al. [3], strengthening damaged cantilever walls with CFRP sheets not only increased the ultimate moment capacity but also improved the overall deformation capacity of the wall system. These enhancements are achieved while maintaining the geometrical and functional integrity of the structure, making CFRP retrofitting a highly practical approach.

Numerous experiments and simulations have confirmed that externally bonded CFRP can dramatically increase the flexural and shear capacity of concrete members. For example, Wang et al. [9] conducted an experimental investigation on reinforced concrete (RC) beams—both simply supported and two-span continuous—strengthened using externally prestressed CFRP tendons. They examined variables such as prestressing level, tendon layout (straight vs. flexural pattern), and the presence of pre-existing cracks. The results revealed substantial gains in ultimate flexural capacity: approximately 160% improvement for straight-line configuration and 170% for flexural arrangement in simply supported beams, and 113% enhancement for continuous beams. Shear capacity also benefited markedly, scaling with prestressing level, and an increase in beam stiffness and key flexural responses near supports was observed. In small-scale tests, Siddika et al. [10] reported that various strengthening configurations yielded 1.7–60.9% increases in ultimate load-carrying capacity compared to control specimens. Full-length U-wrapping was particularly effective for flexural enhancement, while strip U-wrapping alternated with internal stirrups proved advantageous for shear strengthening. Notably, even a single CFRP strip along the soffit—depending on surface preparation—provided an approximate 10% improvement in load capacity. Haroon et al. [11] demonstrated that adding CFRP to shear spans markedly raised shear strength and improved stirrup strain distribution (e.g., bidirectional layouts made the stirrup strain more uniform at a given load). Finite-element models (e.g., ABAQUS simulations calibrated to tests) likewise reproduce these gains, confirming that increased CFRP area and improved bonding lead to higher peak loads and stiffness. Altogether, these results—from experiments on beams and deep walls to FEM parametric studies—highlight the strong, nonlinear influence of CFRP quantity, orientation, and anchorage on structural response.

Because the CFRP strength-gain depends on many interacting factors (beam size, reinforcement, concrete strength, CFRP area/orientation, etc.), traditional design equations are often conservative or insufficiently accurate. This complexity motivates data-driven prediction methods. In particular, artificial neural networks (ANNs) have been applied to learn from experimental/FEM databases and forecast required CFRP reinforcement. For instance, Nguyen et al. [12] proposed a data-driven approach to predict the shear strength of FRP-reinforced concrete beams using an Artificial Neural Network (ANN) model. They compiled a dataset of 125 experimental cases from the literature, incorporating nine key input parameters such as beam geometry (width, effective depth, shear span-to-depth ratio), concrete compressive strength, and properties of both flexural and shear FRP reinforcements (reinforcement ratios, elastic moduli, tensile strength). Through systematic exploration of different ANN architectures and evaluation via statistical measures, the optimal model achieved impressive predictive accuracy: R^2^ ≈ 0.9634 for training and R^2^ ≈ 0.9577 for testing. Additionally, they conducted sensitivity analysis using Monte Carlo simulations (500 runs), which revealed the influence of reinforcement-related variables on model stability and performance. In short, the wealth of experimental/FEM evidence that CFRP greatly boosts RC member strength underpins the use of intelligent ANN-based methods to predict the “optimal” CFRP area or layout for a given performance goal [9,12].

The effectiveness of CFRP systems is also well-documented in industry standards. According to ACI 440.2R-17, externally bonded FRP systems are recommended for increasing the flexural and shear capacities of RC members and improving their ductile behavior, particularly in cases where conventional reinforcement methods are infeasible or cost-prohibitive [13]. Furthermore, their application is considered environmentally and economically favorable due to reduced construction waste, shorter installation times, and minimal energy consumption compared to full structural replacement [2,13]. In light of these advantages, CFRP has become a cornerstone technology in modern structural retrofitting practices—particularly for enhancing the flexural performance of cantilever concrete walls under increased service demands or post-damage rehabilitation scenarios. Designing an optimum CFRP retrofit requires carefully selecting variables such as the number of CFRP layers, strip width, orientation, spacing, and anchorage, so that the strengthened wall just meets (but does not grossly exceed) the required flexural strength. Building codes (ACI 318 for concrete and ACI 440.2R for FRP) provide limit states for flexural and shear capacity that must be satisfied [13,14]. In optimization-based CFRP retrofit design, the total CFRP area (or associated material cost) is commonly chosen as the primary objective to be minimized—reflecting practical concerns over economy—while ensuring that structural strength constraints are satisfied. For example, Rahman et al. [15] formulated an optimization problem for reinforced concrete beams strengthened with externally bonded CFRP plates. They employed a Genetic Algorithm (GA) to minimize the combined cost of CFRP plates and adhesive, subject to serviceability and ultimate limit state constraints derived from the TR55 design guidelines. That study and others confirm that a balanced design can be achieved: using just enough CFRP to satisfy the code-strength requirements. Metaheuristic methods such as GA, JAYA and other are especially well-suited for the non-linear, discrete optimization challenges often encountered in retrofit design. In this context, the Jaya algorithm—introduced by Rao has gained attention for its simplicity and effectiveness. Unlike other metaheuristics, Jaya requires no algorithm-specific control parameters beyond population size and iteration count, and iteratively steers candidate solutions toward the global best and away from the worst alternatives. Studies evaluating Jaya on a suite of benchmark optimization problems have demonstrated that it achieves superior or comparable performance to established algorithms, notably excelling in constrained scenarios [16]. Jaya optimizations can be applied to cantilever walls by substituting moment or shear demands from the wall into the design equations. While metaheuristic optimization finds high-quality CFRP designs, it remains time consuming to run for each new wall geometry or loading case. To accelerate the process, machine learning models (especially Artificial Neural Networks, ANN) can be trained to predict optimum CFRP layouts. Recent studies demonstrate that ANNs can capture the relationship between structural parameters and optimal strengthening needs. For instance, Kayabekir et al. developed an Artificial Neural Network (ANN) model trained on optimal CFRP configurations designed to improve the shear capacity of RC beams. Their approach utilized training datasets derived from metaheuristic-optimized CFRP layouts, enabling the ANN to accurately predict the required CFRP reinforcement ratios and orientations for previously unseen beam scenarios. This strategy demonstrates how ANNs can operationalize computationally intensive optimization results into fast, adaptable design tools [17]. Similarly, other researchers have used ensemble learning and other AI algorithms to predict the capacity of FRP-strengthened members. For example, Zhang et al. introduced an interpretable ensemble learning methodology to predict the flexural capacity of FRP-strengthened reinforced concrete beams, grounded on a comprehensive experimental database. Their study compared various ensemble algorithms and emphasized model transparency—highlighting feature importance and the decision-making rationale of the predictors [18]. A critical examination of the above studies reveals that, while substantial progress has been made in experimental, numerical, and AI-driven approaches for FRP/CFRP retrofit design, important gaps remain. Experimental works (e.g., Wang et al., Siddika et al., Haroon et al.) have established the performance benefits of CFRP in beams and other flexural or shear-critical members, yet their findings are case-specific and do not directly yield generalized design tools. Data-driven models (e.g., Nguyen et al., Kayabekir et al., Zhang et al.) have demonstrated the predictive power of ANN and ensemble learning for FRP-strengthened members, but have primarily targeted beams and focused on capacity estimation rather than direct optimization of CFRP usage. Optimization-oriented studies (e.g., Rahman et al.) have minimized CFRP cost or area under code-based constraints but without coupling these algorithms to predictive AI models for rapid deployment. The present research addresses these limitations by focusing on RC cantilever shear walls—a structural configuration seldom treated in previous optimization–AI hybrid studies—and by integrating a metaheuristic optimizer (Jaya algorithm) with an ANN surrogate model to deliver instantaneous, near-optimal CFRP layouts for flexural strengthening.

Inspired by these findings, the present work develops an ANN-based predictor for CFRP design in cantilever shear walls. We collect optimized design data from the Jaya algorithm—generated under varying wall geometry, material properties, and loading conditions—and use a Levenberg–Marquardt backpropagation ANN to learn the underlying mapping between problem inputs and optimal CFRP configurations. Once trained, the ANN can instantaneously suggest near-optimal CFRP layouts for new design cases without running a fresh optimization. In summary, the proposed hybrid framework leverages the accuracy of metaheuristic optimization and the speed of AI prediction, providing a rapid, accurate, and code-compliant design tool for flexural strengthening of RC cantilever shear walls.

In addition, to situate the proposed method within the broader research landscape, Table 1 provides a comparative summary of recent high-impact studies, contrasting their scope, methodology, and contributions with those of the present work.

## 2. Materials and Methods

### 2.1. JAYA Algorithm

The Jaya algorithm, proposed by Rao [16], is a population-based metaheuristic optimization technique inspired by the concept of victory—”Jaya” in Sanskrit. The core idea is to guide solutions toward the best candidate and away from the worst. Unlike many evolutionary algorithms, Jaya is parameter-free, requiring only the population size and number of iterations, which makes it simple and user-friendly [19,20]. In each iteration, every solution is updated using the best and worst candidates in the population.

The update rule is defined as

x_i,j_^t+1^ = x_i,j_^t^ + r_1_ (x_i_* − |x_i,j_^t^|) − r_2_ (x_i_^w^ − |x_i,j_^t^|) i = 1, 2, …, n; j = 1, 2, …, p; t = 1, 2, …, t_max_ (1)

where x_i,j_^t^ is the i-th variable of the j-th solution at iteration t; x_i_* and x_i_^w^ are the best and worst values for that variable across the population; and r_1_,r_2_∈ [0, 1] are randomly generated coefficients. The algorithm starts by generating an initial population within the predefined design variable bounds:

x_i_ ^j^ = x_i min_ + rand (1) (x_i max_ − x_i min_) i = 1, 2, …, n and j = 1, 2, …, p (2)

Each candidate is evaluated using an objective function and updated iteratively until a stopping criterion is met. Updated variables are adjusted if they exceed bounds. This simple structure avoids the complexity of tuning multiple parameters, such as crossover or inertia weights in GA or PSO [21]. By simultaneously promoting convergence toward the optimal solution and avoiding poor regions in the design space, Jaya maintains a healthy balance of exploration and exploitation. This helps reduce the risk of being trapped in local optima, enhancing its applicability to a variety of complex engineering problems [22]. The algorithm’s robustness and simplicity have led to its successful application in numerous civil engineering tasks. For instance, Grzywiński et al. [23] applied the Jaya algorithm to optimize the design of a 2D reinforced concrete frame (three-bay, three-story) under Turkish code provisions, achieving cost-effective design solutions. Similarly, Aslay and Dede [24] demonstrated a 3D optimization of a three-story reinforced concrete structure with shear walls and frames using ETABS (v14)-MATLAB (v2021) integration, effectively reducing construction costs. Furthermore, Phan and Phan [25] utilized Jaya optimization to minimize material usage (concrete and reinforcement) in a 3D RC frame, achieving robust and fast convergence via a Python (v3.10)-ETABS (v19) workflow. In cross-sectional optimization, Coşut [26] applied Jaya to size RC rectangular beams under different codes (ACI 318, TS500, Eurocode 2), achieving cost savings up to 8% by minimizing material quantities. Duysak et al. [21] used Jaya for combined torsion–shear–flexure design of slab beams under ACI 318, optimizing section dimensions and reinforcement for minimum cost. Their results confirmed the algorithm’s reliability under multiple loading conditions. In another study, Eroğlu et al. [27] applied Jaya to cantilever retaining wall design under both static and seismic loading. The algorithm effectively minimized the cost per unit length while satisfying safety limits for sliding, overturning, and bearing capacity based on Turkish seismic code (DBYBHY-2007), reducing total costs by up to 21%. These examples confirm that Jaya can address diverse tasks in structural engineering, such as section sizing, reinforcement optimization, and strengthening strategies. Its strength lies in being both efficient and adaptable to practical design constraints, especially when computational simplicity and reliable convergence are desired [28].

In this study, the Jaya algorithm was employed to utilize a dataset of 500 optimized CFRP retrofitting scenarios for cantilever concrete walls. Each scenario was defined by a unique set of input parameters Table 2, including wall geometry, axial load, concrete and steel properties, CFRP material properties, and flexural demand. The python code which generates 500 scenarios (rows) of input parameters for JAYA is provided in Table 3.

The range of design variables used in the optimization (Table 2) reflects values found in established standards, such as ACI 318 and ACI 440.2R-17 [13,14] and published literature. For instance, the adopted ranges for wall dimensions, reinforcement ratios, and CFRP properties are consistent with those presented in experimental studies by El-Kashif et al. [29] and Altin et al. [30]. The dataset used to train the ANN was generated synthetically using the Jaya algorithm, with constraints aligned with real-world design parameters observed in engineering practice. This ensures that the resulting predictions are grounded in feasible and code-compliant configurations.

Based on the provided inputs, for each input row, the JAYA algorithm aimed to determine the values of the optimal set of design variables (i.e., of 3 variables)—namely the number of CFRP layers (n), strip width (W_f_), and thickness (t_f_)—that would satisfy the flexural strength requirement while minimizing the total CFRP area as the objective function, which is defined as:

A_CFRP_ = 2 × n × W_f_ × t_f_ (3)

All design variables are bounded by practical limits to ensure feasible CFRP layouts according to Equations between Equation (4) and Equation (6)

1 ≤ n ≤ 5(4)

100 ≤ W_f_ ≤ 500 (5)

0 ≤ t_f_ ≤ 1.5 (6)

One constraint enforces the strength requirement according to ACI 318R-05 and ACI 440.2R-2017 as shown in Equation (7):

g = ϕM_n_ − M_u_ > 0 (7)

The factored nominal moment capacity (ϕM_n_) must be greater than the applied ultimate moment (M_u_), ensuring a positive safety margin, as expressed by the inequality. The resulting 500 optimized designs formed the basis for training an Artificial Neural Network (ANN) model, which learned to predict near-optimal CFRP configurations from the given structural parameters. Additionally, five separate test cases were generated using the same framework but were excluded from training as shown in Table 4 These were used as a benchmark to assess the ANN’s predictive accuracy in estimating the optimal CFRP layout for new, unseen structural conditions.

### 2.2. Artificial Neural Network

Artificial Neural Networks (ANNs) are computational frameworks inspired by the biological neural networks found in human brains. These systems are designed to recognize patterns and extract complex relationships from data by simulating interconnected processing nodes, known as neurons. Each artificial neuron receives weighted inputs, applies an activation function, and transmits the result to the subsequent layer. The learning process of ANNs involves adjusting the weights based on error minimization through backpropagation algorithms [31].

The concept of ANNs traces back to the 1940s, with the introduction of the perceptron by McCulloch and Pitts in 1943 [32], who modeled a simple neuron as a binary threshold unit. The perceptron laid the groundwork for neural computation by demonstrating how interconnected nodes could process inputs to produce outputs. Nevertheless, the initial models demonstrated significant limitations in addressing complex, non-linear problems—a shortcoming critically examined by Minsky and Papert [33] in their 1969 evaluation of single-layer perceptrons. The resurgence of ANNs in the 1980s was driven by the development of backpropagation, a gradient-based optimization technique introduced by Rumelhart, Hinton, and Williams in 1986 [34]. Backpropagation enabled multi-layer perceptrons (MLPs) to learn complex patterns by adjusting connection weights based on error gradients. This breakthrough addressed the limitations of earlier models and paved the way for deep learning, a subset of ANNs characterized by multiple hidden layers [35]. ANNs are grounded in the principle of distributed processing, where interconnected nodes (neurons) work collaboratively to solve problems. Each neuron processes input data, applies a weighted sum, and passes the result through an activation function to produce an output. This structure mimics synaptic connections in biological brains, enabling ANNs to model non-linear relationships [36]. The architecture of an ANN comprises three primary layers: input, hidden, and output. The input layer receives raw data, such as pixel values in image recognition tasks. Hidden layers, which may vary in number and size, perform feature extraction and transformation through weighted connections. The output layer produces the final prediction or classification [37]. Several types of ANN architectures exist, each suited to specific tasks:

1. Feedforward Neural Networks (FNNs): These are the simplest ANNs, where information flows unidirectionally from input to output. FNNs are widely used for tasks like regression and classification [36].
2. Convolutional Neural Networks (CNNs): These are a specialized type of ANN primarily utilized in image-related tasks. By applying a series of convolutional filters across the input data, CNNs are capable of capturing local spatial hierarchies and patterns. This architectural feature makes them particularly well-suited for applications such as image classification, object detection, and visual recognition tasks [38].
3. Recurrent Neural Networks (RNNs): These networks are designed to handle sequential and time-dependent data by incorporating internal memory. Unlike traditional feedforward networks, RNNs retain information from previous steps in the sequence, enabling them to model temporal behaviors in tasks like speech recognition, time series prediction, and natural language processing [39].
4. Deep Neural Networks (DNNs): These are ANNs with multiple hidden layers, enabling the modeling of complex, hierarchical patterns. DNNs underpin advancements in deep learning [35].

Each architecture employs activation functions, such as sigmoid, ReLU (Rectified Linear Unit), or tanh, to introduce non-linearity, allowing ANNs to solve complex problems [37].

ANNs learn by adjusting the weights of connections between neurons to minimize prediction errors. This process, known as training, typically involves supervised, unsupervised, or reinforcement learning paradigms:

- Supervised Learning: Involves training ANNs on labeled datasets, where the model learns to map inputs to known outputs. Backpropagation, combined with optimization algorithms like stochastic gradient descent (SGD), is commonly used [34].
- Unsupervised Learning: ANNs identify patterns in unlabeled data, often through techniques like autoencoders or clustering. This is useful for tasks like dimensionality reduction [40].
- Reinforcement Learning: ANNs learn by interacting with an environment, optimizing actions based on rewards. Deep reinforcement learning, as exemplified by AlphaGo, integrates ANNs with reinforcement learning principles [41].

Training ANNs requires large datasets and computational resources. Techniques like regularization, dropout, and batch normalization mitigate overfitting and improve generalization [42]. Hyperparameter tuning, such as adjusting learning rates or layer sizes, further enhances performance [37].

ANNs have transformed numerous domains due to their versatility and ability to model complex patterns:

- Computer Vision: CNNs excel in tasks like image classification, object detection, and facial recognition. For instance, AlexNet, a deep CNN, achieved groundbreaking results in the 2012 ImageNet competition [43].
- Natural Language Processing (NLP): RNNs and transformer-based models, which build on ANN principles, power applications like machine translation, sentiment analysis, and chatbots [44].
- Healthcare: ANNs analyze medical images, predict disease outcomes, and personalize treatment plans. For example, DNNs have been used to detect diabetic retinopathy with high accuracy [45].
- Autonomous Systems: ANNs enable self-driving cars and robotics by processing sensor data for navigation and decision-making [46].
- Finance: ANNs predict stock prices, detect fraud, and assess credit risk by analyzing complex datasets [47].
- Civil Engineering: In civil engineering, ANNs are transformative for optimizing structural designs and predicting material behaviors. For instance, Seyed Hakim et al. [48] developed a multilayer feedforward neural network (MFNN) trained on 368 mix design samples using eight input parameters including cement, water, aggregates, and mineral admixtures. Their optimal network architecture (8-10-6-1) achieved high accuracy with a relative percentage error of 7.02% in training and 12.64% in testing, demonstrating the practicality of ANNs for modeling nonlinear behavior in HSC mixtures.

These applications highlight the adaptability of ANNs across diverse fields, driven by advancements in computational power and data availability.

Despite their success, ANNs face several challenges:

- Computational Complexity: Training deep ANNs requires significant computational resources, limiting accessibility for smaller organizations [35].
- Overfitting: ANNs may memorize training data rather than generalize, necessitating techniques like dropout and regularization [42].
- Interpretability: ANNs are often described as “black boxes” due to their complex internal workings, posing challenges for trust and accountability in critical applications [49].
- Data Dependency: ANNs require large, high-quality datasets, which may not always be available, particularly in specialized domains [37].
- Ethical Concerns: Bias in training data can lead to unfair or discriminatory outcomes, as seen in some facial recognition systems [50].

Addressing these challenges requires ongoing research into efficient algorithms, interpretable models, and ethical frameworks.

The future of ANNs lies in improving efficiency, interpretability, and robustness. Techniques like transfer learning and few-shot learning aim to reduce data dependency, while explainable AI seeks to enhance transparency [49]. Neuromorphic computing, which mimics biological neural structures more closely, promises energy-efficient ANNs [51]. Additionally, integrating ANNs with quantum computing could unlock new computational paradigms [52]. In this study, a feedforward artificial neural network (ANN) was constructed to estimate the optimum carbon fiber reinforced polymer (CFRP) area required to enhance the flexural capacity of reinforced concrete (RC) shear walls. The model development and training were carried out using the Neural Net Fitting Tool (nftool) available in MATLAB, which is specifically tailored for function approximation and nonlinear regression problems.

**Figure 2:**
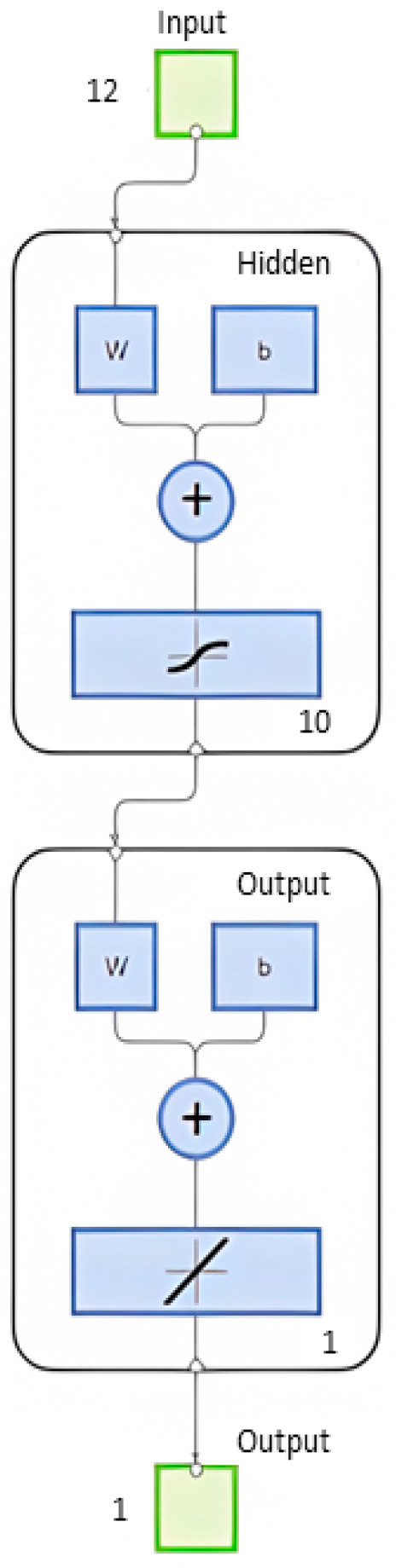
Schematic representation of the ANN structure with 12 input neurons, 10 hidden neurons, and 1 output neuron (A_f_). The letters ‘w’ and ‘b’ represent the synaptic weights and biases, respectively, while the numbers indicate the size of each layer.

**Figure 3:**
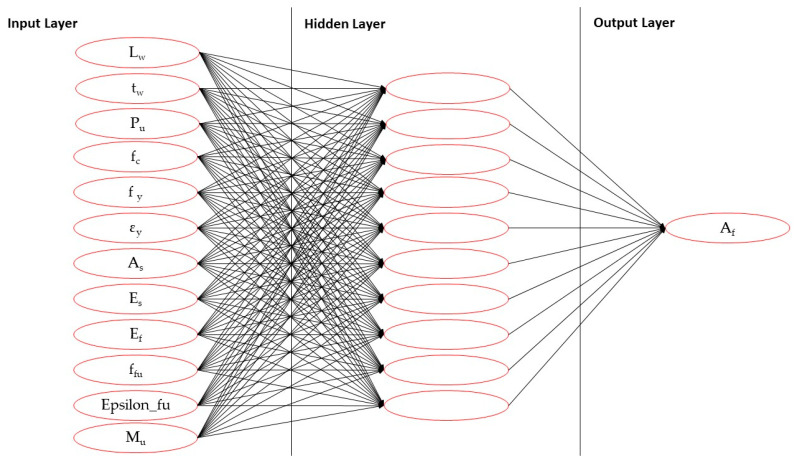
Schematic architecture of the artificial neural network (ANN) model.

The ANN model adopted a multilayer perceptron (MLP) structure composed of three layers: an input layer, one hidden layer, and an output layer. As shown in Figure 2 and Figure 3 a total of 12 input parameters were defined based on the physical and mechanical properties of the RC element and CFRP material, was provided in Table 2 with their ranges:

The network output was defined as the optimum CFRP area (A_f_) necessary to meet the flexural strength requirement with minimum material usage. The hidden layer consisted of 10 neurons, and the transfer functions used were the tansig (hyperbolic tangent sigmoid) function in the hidden layer and the purelin (linear) function in the output layer. This configuration was determined through iterative testing, balancing training accuracy and generalization ability.

The fundamental building blocks of ANNs are artificial neurons. A neuron multiplies inputs with weights, adds a bias, and produces an output via an activation function. Mathematically, the output of a neuron is expressed as shown in Equation (8):

(8) $$y=f\;(\sum_{i=1}^{n}Wi\times Xi+b)$$

where Xi are the inputs; Wi are the weights; b is the bias; and f is the activation function. Activation functions enable neurons to perform nonlinear transformations, allowing the network to model complex relationships [53].

The ANN was trained using the Levenberg–Marquardt (LM) backpropagation algorithm, which is known for its rapid convergence and robustness in handling nonlinear problems. The LM algorithm is especially effective for small-to-medium sized datasets and is widely used in engineering applications due to its numerical stability.

To evaluate the model’s learning performance, the mean squared error (MSE) was employed as the primary performance metric. MSE calculates the average of the squared differences between the actual and predicted outputs, offering a clear measure of predictive accuracy. It is defined mathematically as follows in Equation (9):

(9) $$\mathrm{MSE}=\frac{1}{n}\sum_{i=1}^{n}{\left(yi-y^{\prime }i\right)}^{2}$$

where yi is the actual target value, y′i is the corresponding output predicted by the ANN, and n is the number of samples [54].

The dataset was randomly divided into three subsets to facilitate supervised training and to ensure the model’s generalization:

- Training set: 70% of the data, used to update the network’s weights and biases through iterative learning.
- Validation set: 15%, used to monitor the model’s performance during training and to prevent overfitting via early stopping.
- Testing set: 15%, used to assess the model’s predictive capability on unseen data and ensure its robustness.

**Figure 4:**
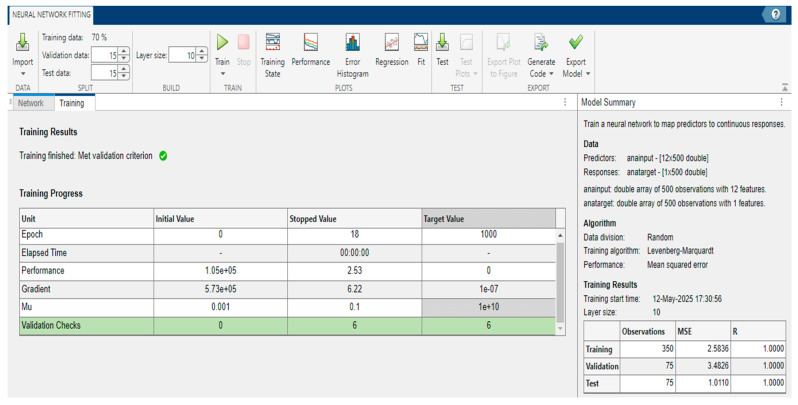
Data division and training setup using Levenberg–Marquardt algorithm.

This partitioning strategy is illustrated in Figure 4, demonstrating a standard approach used in ANN-based regression models.

In addition, a 10-fold cross-validation (CV) was also implemented following this stage with a similar ANN architecture/model using the Regression Learner app in MATLAB with a Training/Validation data split of 10 × (90–10%), utilizing a narrow ANN architecture with a single hidden layer containing 10 neurons. The results of the both training and evaluation stages are provided in the next section.

In summary, a single-hidden-layer feedforward ANN architecture with 10 neurons was implemented and trained using the Levenberg–Marquardt backpropagation algorithm. The model showed excellent convergence and low training error, validating its effectiveness for predicting CFRP strengthening demands in RC walls. Its simplicity, speed, and accuracy make it well-suited for integration into design workflows.

## 3. Results and Discussion

This section presents and analyzes the performance of the Artificial Neural Network (ANN) model developed to predict the optimum Carbon Fiber Reinforced Polymer (CFRP) area required for enhancing the flexural strength of reinforced concrete (RC) cantilever shear walls. The ANN was trained on a dataset of 500 optimized CFRP retrofitting scenarios generated using the Jaya algorithm, with its predictive accuracy assessed on five unseen test cases. The results are presented and analyzed in the following subsections, providing a detailed evaluation of the model’s regression performance, error distribution, training dynamics, and practical applicability.

### 3.1. Regression Analysis and Model Accuracy

The predictive performance of the developed ANN model was initially assessed using the coefficient of determination (R) and the mean squared error (MSE) across the training, validation, and testing datasets. As presented in Table 5, the model achieved perfect correlation (R = 1.0) for the training, validation, and test sets, indicating excellent fitting and generalization. Additionally, a 10-fold cross-validation (CV) was conducted to further confirm the robustness of the implemented approach yielding a mean R-value of 0.9996 with a higher mean MSE due to the broader data range of the validation set.

**Figure 5:**
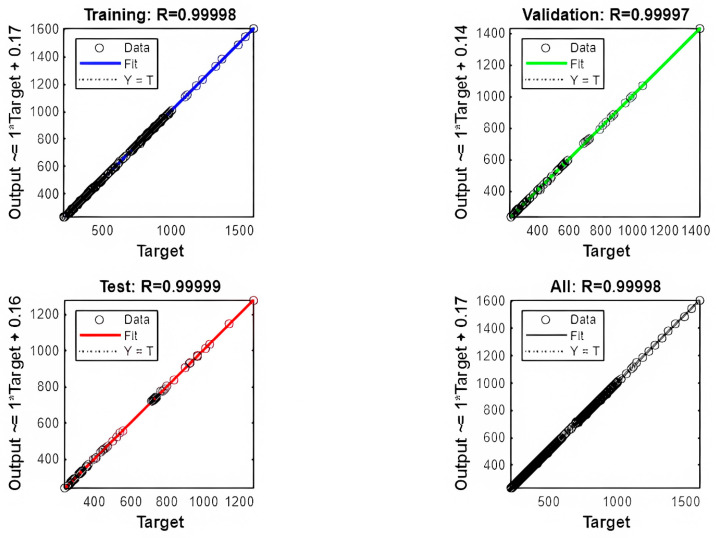
Targets vs. outputs in training/validation/test sets.

**Figure 6:**
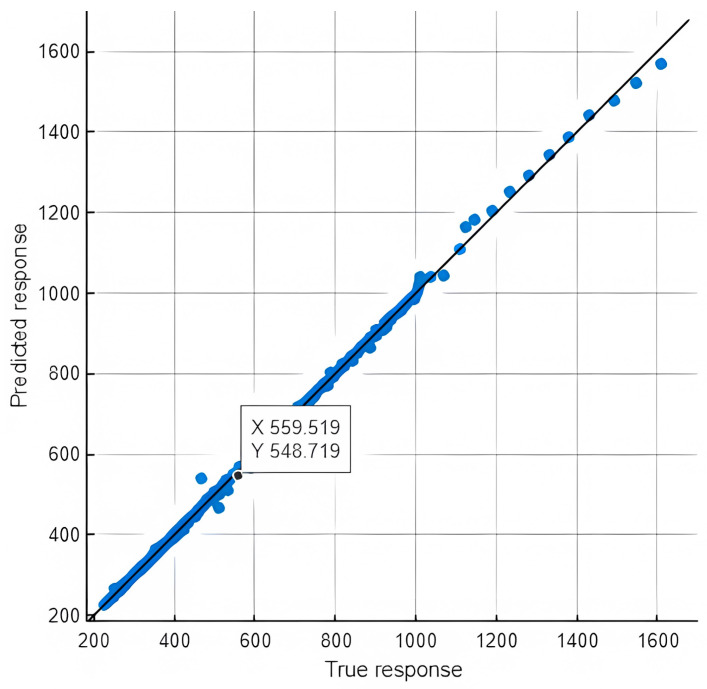
Predicted vs. actual plot of 10-fold cross-validation (comparing ANN-predicted and Jaya-optimized CFRP areas).

These outcomes are visually corroborated in Figure 5 (Target vs. Output Plots) and Figure 6 (Predicted vs. Actual Plot), which graphically validate the near-perfect alignment between predicted and optimized CFRP areas across all evaluation phases.

The remarkably high correlation coefficients across all data subsets highlight the ANN model’s strong predictive performance. The alignment between the predicted A_f_ values and those obtained from the Jaya optimization underscores the machine learning model’s ability to accurately capture the underlying design relationships. This consistency reflects the network’s robustness and reliability when applied to new, unseen input configurations, an essential feature for real-world structural engineering applications.

### 3.2. Error Distribution and Validation

**Figure 7:**
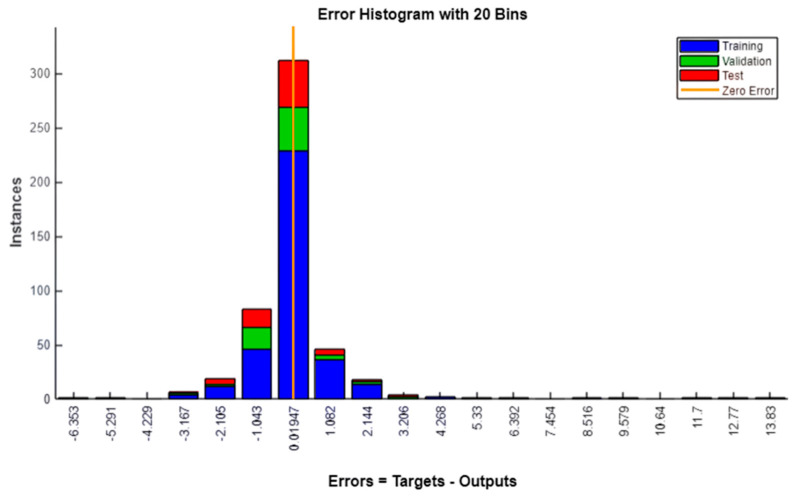
Error histogram with 20 bins.

**Figure 8:**
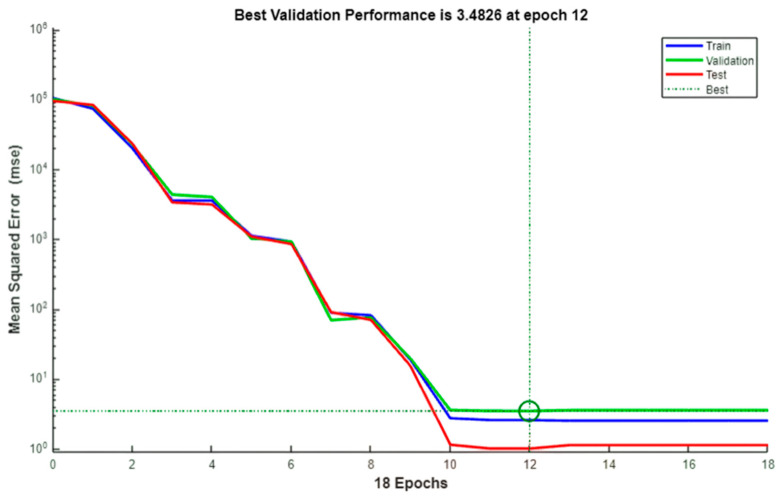
Best validation performance plot (MSE vs. Epoch). The circle indicates the epoch at which the best validation performance was achieved.

To further assess prediction accuracy, an error histogram was generated, as shown in Figure 7. The histogram, constructed with 20 bins, illustrates that the majority of prediction errors cluster tightly around zero, with only minor deviations. This distribution underscores the precision of the ANN model, as it consistently delivers predictions closely aligned with the target values. The training process was monitored using the MSE, with Figure 8 plotting the best validation performance over epochs. The rapid initial decrease in validation MSE, followed by stabilization, indicates efficient convergence and effective learning. The training halted when the validation error ceased to improve, a strategy implemented via early stopping to avoid overfitting and ensure robust generalization.

### 3.3. Training Dynamics

**Figure 9:**
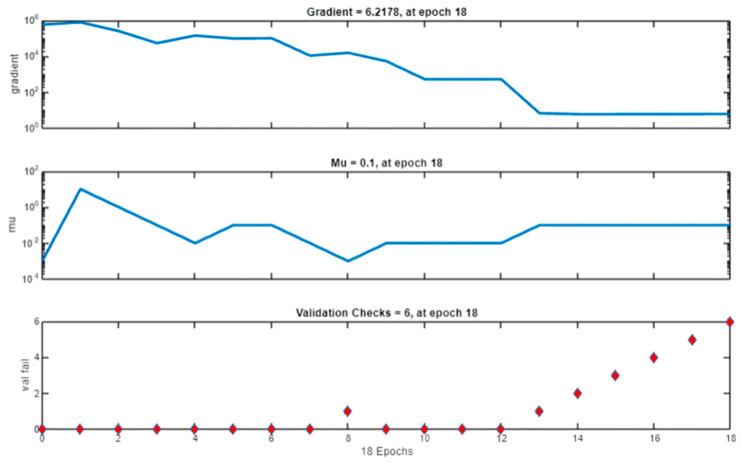
Gradient, Mu, and Validation Checks. The blue lines represent the values of the gradient and learning rate (Mu) over epochs, while the red markers in the bottom plot indicate the validation failure counts.

Figure 9 provides insights into the training dynamics, displaying the gradient, the adaptive learning parameter (m_u_), and validation checks. The gradient decreases steadily, reflecting the model’s progress in minimizing the error surface. The (m_u_) parameter, integral to the LM algorithm, adjusts dynamically to optimize step sizes, while the validation checks confirm that training stopped at an appropriate point, balancing accuracy and computational efficiency.

### 3.4. Predictive Performance on Unseen Data

The generalization capability of the developed ANN model was assessed using five independent cases that were generated via the Jaya algorithm but excluded from the training dataset. Table 6 presents a comparison between the optimum CFRP areas (A_f_) obtained from the Jaya optimization and the corresponding predictions made by the ANN, along with the computed percent errors.

The ANN model achieved an average percent error of 3.69%, demonstrating strong predictive accuracy across all five test cases. Notably, four of the five cases (Cases 2, 3, 4, and 5) yielded errors below 6%, reflecting the model’s robustness and generalization capability. Even in Case 1, which exhibited the highest deviation of 10.11%, the prediction remains within acceptable engineering limits. These results confirm the ANN’s effectiveness in estimating optimal CFRP reinforcement areas for diverse design scenarios.

### 3.5. Global Sensitivity Analysis via Perturbation Method

To evaluate the global importance of each input parameter on the predicted CFRP area, a perturbation-based global sensitivity analysis was conducted. This method operates by systematically varying each input variable across its entire range (as defined by the dataset) while keeping all other variables constant at their baseline values (typically the mean or median). The resulting changes in the model output reflect the global influence of the individual feature.

**Figure 10:**
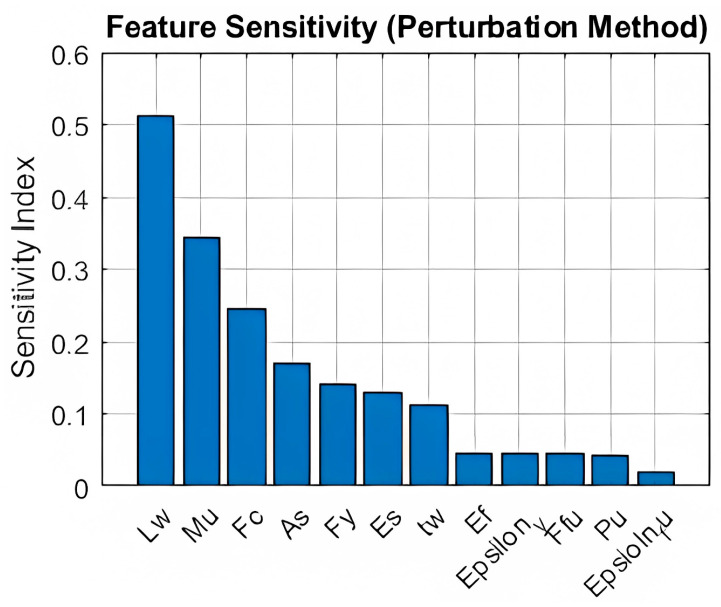
Feature Sensitivity Analysis using Perturbation Method.

This technique captures nonlinear interactions and provides a comprehensive measure of the overall importance of each design variable on the output. Figure 10 presents the sensitivity indices for all input features.

As shown in Figure 10, wall length (L_w_), moment demand (M_u_), and concrete compressive strength (F_c_) were identified as the most influential parameters. These insights are essential for guiding engineers toward the most impactful variables in the design and strengthening of RC shear walls with CFRP.

### 3.6. Discussion and Implications

The hybrid ANN–Jaya framework developed in this study leverages an ANN model trained on 500 Jaya-optimized CFRP strengthening scenarios to deliver rapid, reliable design predictions. By learning the complex mapping between twelve input parameters (wall geometry, applied loads, and material properties of concrete, steel, and CFRP) and the optimal CFRP area, the network achieves an average prediction error of 3.69% on five unseen cases. This level of accuracy substantiates its integration into a streamlined design methodology for CFRP-retrofitted RC cantilever walls.

In practical application, the engineer first assembles the full set of twelve design inputs—wall length and thickness, axial load and ultimate moment demand, concrete compressive strength, steel yield strength and strain, reinforcement area and modulus, and CFRP modulus, tensile strength, and strain. These inputs are then fed into the ANN, which instantly returns the required CFRP area A_f_, _ANN_. Compliance with ACI 318 and ACI 440.2R-17 is verified by confirming that the factored nominal moment capacity ϕM_n_ exceeds the specified ultimate moment M_u_, thereby ensuring both safety and code conformity.

To illustrate the sensitivity of CFRP demand to moment loading and other parameters, the results of the global sensitivity analysis (Section 3.5) offer valuable insights. For example, wall length (L_w_), moment demand (M_u_), and concrete compressive strength (F_c_) demonstrated the highest influence on the predicted CFRP area. These findings highlight the ANN model’s capacity to capture nonlinear relationships and identify the most critical design variables.

Additionally, Table 4 presents five representative cases generated by the Jaya algorithm. These cases span ultimate moment demands Mu from 330 kN·m to 405 kN·m, with corresponding optimal CFRP areas A_f_ varying between 393.62 mm^2^ and 630.67 mm^2^. For example, increasing M_u_ from 330 kN·m to 380 kN·m (a 15% rise) leads to an increase in Af from 461.86 mm^2^ to 630.67 mm^2^ (a 36% rise). A further increase to 405 kN·m (23% above baseline) yields A_f_ = 538.38 mm^2^. These data reinforce the model’s responsiveness to input variation.

Beyond rapid prediction, this approach confers several tangible benefits. First, the reduction in iterative optimization runs frees computational resources and accelerates project timelines. Second, the close agreement between ANN outputs and Jaya-derived optima (within 4% on average) ensures that designs remain both economical and conservative. Third, the framework’s modular nature allows straightforward extension to other structural forms—such as beams, slabs, or columns—by retraining the network on appropriately curated datasets.

Finally, embedding the ANN predictions within a formalized design procedure aligns with modern performance-based engineering and sustainability objectives. By minimizing unnecessary CFRP usage, the approach reduces material costs and the environmental footprint of retrofits, without compromising structural safety. Future enhancements might integrate multi-objective criteria—balancing cost, carbon impact, and performance—or incorporate uncertainty quantification to address variability in material properties and loading. Nevertheless, this work establishes a clear, validated pathway for deploying ANN-driven predictions as the centerpiece of CFRP strengthening design, offering a consolidated, parametric, and practically grounded methodology.

## 4. Conclusions

This paper has presented a novel, data-driven methodology that integrates a multilayer perceptron artificial neural network (ANN) with the Jaya metaheuristic optimization algorithm to efficiently design CFRP strengthening solutions for RC cantilever shear walls. The ANN was trained on 500 Jaya-derived optimal designs covering a comprehensive range of twelve input parameters—encompassing geometric dimensions, loading conditions, and material properties of concrete, steel, and CFRP. Validation against five independent test cases yielded an average percent error of 3.69%, confirming the model’s strong generalization capability and reliability for unseen design scenarios.

Key findings include the following:

- High Predictive Accuracy: The ANN consistently reproduced Jaya-optimized CFRP areas with errors below 11% in the worst case and below 6% in four of five scenarios, demonstrating that machine learning can supplant iterative optimization with minimal loss of precision.
- Global Sensitivity Insights: Through a perturbation-based sensitivity analysis, wall length (L_w_), moment demand (M_u_), and concrete compressive strength (F_c_) were identified as the most influential factors on CFRP demand. This analysis enhances the interpretability of the model and informs engineers on which parameters most critically affect retrofitting requirements.
- Parametric Sensitivity: A focused study on moment demand showed that a 15% increase in ultimate moment led to a 36% rise in required CFRP area, highlighting the model’s ability to capture nonlinear interactions and enabling rapid “what-if” analyses.
- Design Workflow Integration: Embedding the trained ANN into a streamlined design procedure transforms retrofit design from cumbersome trial-and-error into an instantaneous prediction step, where twelve readily available inputs yield a code-verified CFRP area that satisfies ACI 318 and ACI 440.2R-17 requirements.

Beyond computational efficiency, the proposed framework advances sustainable engineering practice by minimizing unnecessary CFRP usage—thereby reducing material costs and environmental impact—while upholding structural safety. Its modular architecture facilitates extension to other structural elements (such as beams, slabs, and columns) through retraining on element-specific datasets.

Future work should explore multi-objective formulations that concurrently optimize CFRP area, lifecycle cost, and carbon footprint, as well as incorporate uncertainty quantification to address variability in materials and loading. Moreover, coupling this ANN-based approach with user-friendly software interfaces or APIs could accelerate adoption in engineering practice.

In summary, this study establishes a validated, practically grounded methodology in which ANN-driven predictions serve as the centerpiece of CFRP strengthening design—delivering rapid, accurate, and resource-efficient solutions for modern structural retrofitting challenges.

## Figures and Tables

**Table 1:** Comparative summary of recent high-impact studies on FRP/CFRP strengthening.

Ref.	Structural Element Type	FRP Configuration	Modeling/Approach	Optimization/AI Algorithm	Performance Metric	Key Contribution
Wang et al. (2024) [9]	RC beams (simply supported, continuous)	Prestressed CFRP tendons (straight and flexural layouts)	Experimental testing	–	% increase in flexural and shear capacity	Quantified flexural and shear gains with different prestressing levels and layouts
Siddika et al. (2019) [10]	RC beams	Full-length U-wrap, strip U-wrap, soffit strips	Experimental testing	–	% increase in ultimate load	Compared CFRP layouts for flexural vs. shear enhancement
Haroon et al. (2021) [11]	RC beams	CFRP strips in shear spans (unidirectional, bidirectional)	Experimental testing	–	Shear strength increase, stirrup strain uniformity	Demonstrated improved shear behavior and strain distribution
Nguyen et al. (2021) [12]	RC beams	Flexural and shear FRP	ANN prediction model	ANN (multiple architectures)	R^2^, sensitivity analysis	Predicted shear strength with high accuracy from experimental database
Rahman et al. (2012) [15]	RC beams	Externally bonded CFRP plates	Analytical design + GA optimization	Genetic Algorithm	Minimized CFRP cost	Optimized CFRP layout under TR55 constraints
Kayabekir et al. (2019) [17]	RC beams	CFRP for shear strengthening	ANN trained on metaheuristic-optimized data	ANN	Prediction accuracy	Predicted optimal CFRP ratios/orientations from training data
Zhang et al. (2022) [18]	RC beams	FRP strengthening (flexural)	Ensemble learning model	Gradient boosting, etc.	R^2^, feature importance	Interpretable AI model predicting flexural capacity
Present study	RC cantilever shear walls	CFRP for flexural strengthening	ANN surrogate trained on optimization results	Jaya + ANN	Minimized CFRP area, prediction error (%)	Hybrid optimization–AI framework for rapid, code-compliant retrofit design

**Table 2:** Input parameters and ranges.

Parameter	Symbol	Range
Wall length	L_w_	1100–1600 mm
Wall thickness	t_w_	100–220 mm
Axial load	P_u_	36–75 kN
Concrete compressive strength	f_c_	16–30 MPa
Steel yield strength	f_y_	255–400 MPa
Reinforcement yield strain	ε_y_	0.001–0.0019
Reinforcement area	A_s_	450–940 mm^2^
Modulus of elasticity of steel	E_s_	185,000–234,000 MPa
Modulus of elasticity of CFRP	E_f_	65,100–70,000 MPa
CFRP ultimate tensile strength	f_fu_	805–1050 MPa
CFRP ultimate strain	ε_fu_	0.0106–0.0125
Ultimate moment demand	M_u_	301,000–409,000 N·mm

**Table 3:** Code for generation of 500 scenarios.

def generate_data(): data = [] for thickness in range(1100, 1610, 10): data.append([thickness, 150, 53.4, 17.23, 275.8, 0.0014, 500, 200000, 66200, 917, 0.0114, 348000]) for length in range(100, 225, 5): data.append([1500, length, 53.4, 17.23, 275.8, 0.0014, 500, 200000, 66200, 917, 0.0114, 348000]) for load in range(36, 76): data.append([1250, 150, load, 17.23, 275.8, 0.0014, 500, 200000, 66200, 917, 0.0114, 348000]) for strength in range(16, 31): data.append([1250, 140, 50, strength, 275, 0.0014, 500, 200000, 66200, 917, 0.0114, 348000]) for yield_str in range(255, 405, 5): data.append([1500, 140, 50, 20, yield_str, 0.0014, 500, 200000, 66200, 917, 0.0114, 348000]) for i in range(10): strain = round(0.001 + i * 0.0001, 4) data.append([1500, 140, 50, 20, 300, strain, 500, 200000, 66200, 917, 0.0114, 348000]) for area in range(450, 950, 10): data.append([1300, 130, 55, 18, 300, 0.0014, area, 200000, 66200, 917, 0.0114, 348000]) for modulus in range(185000, 235000, 1000): data.append([1200, 120, 45, 22, 325, 0.0014, 550, modulus, 66200, 917, 0.0114, 348000]) for modulus in range(65100, 70100, 100): data.append([1400, 125, 60, 23, 340, 0.0014, 525, 200000, modulus, 917, 0.0114, 348000]) for strength in range(805, 1051, 5): data.append([1450, 155, 55, 19, 340, 0.0014, 490, 190000, 68000, strength, 0.0114, 348000]) for i in range(20): strain = round(0.0106 + i * 0.0001, 4) data.append([1500, 150, 55, 20, 350, 0.0014, 500, 190000, 69000, 900, strain, 348000]) for moment in range(301000, 410000, 1000): data.append([1600, 145, 50, 18, 270, 0.0014, 520, 195000, 70000, 950, 0.0114, moment]) return data generate_data()

**Table 4:** Optimal values obtained from JAYA algorithm for ANN training.

Data												Optimal Value
L_w_	t_w_	P_u_	F_c_	F_y_	ε_y_	A_s_	E_s_	E_f_	F_fu_	ε_fu_	M_u_	A_f_
1600	150	50	18	275	0.0014	500	200,000	66,900	850	0.0118	400,000	558.83
1500	145	45	17.4	275	0.0014	520	195,000	69,300	1005	0.0114	380,000	630.67
1550	160	51	16.5	280	0.0013	550	198,000	69,500	830	0.0123	350,000	393.62
1400	150	44	25	310	0.0014	512	200,000	69,923	887	0.0125	405,000	538.38
1350	140	56.6	17.7	300	0.0013	570	199,000	68,729	919	0.0114	330,000	461.86

**Table 5:** Performance metrics (MSE and R-value) of the ANN model for training, validation, testing, and 10-fold cross-validation phases.

	Observations	MSE	R
Training Set	350	2.5836	0.9999
Validation Set	75	3.4826	0.9999
Test Set	75	1.0110	0.9999
Validation Set (10-fold CV)	500 *	41.104 **	0.9996 **

* sum of 10-folds ** mean of 10-folds.

**Table 6:** Predictions on new cases results of the ANN model trained on a dataset of 500 samples.

	Case	Optimum Results	ANN Predictions	Percent Error (%)
A_f_ (mm^2^)	Case 1	558.83	615.33	10.11
	Case 2	630.67	639.03	1.33
	Case 3	393.62	391.42	0.56
	Case 4	538.38	570.43	5.95
	Case 5	461.86	464.13	0.49
	Average Percent Error: 3.69%			

## Data Availability

The raw data supporting the conclusions of this article will be made available by the authors on request.
